# Penile cancer in French Guiana: Epidemiology, histopathology and clinical aspects

**DOI:** 10.1002/bco2.70096

**Published:** 2025-10-14

**Authors:** Khalil Chalhoub, Bawa Nonoa, Vincent Molinier, Vincent Ravery

**Affiliations:** ^1^ Service d'Urologie CHU Guyane, Site De Kourou Kourou Guyane Française; ^2^ Service d'Urologie CHU Martinique Fort‐De‐France Martinique; ^3^ Service d'Anatomopathologie CH Du Pays d'Aix Aix En Provence France

**Keywords:** circumcision, French Guiana, human papilloma virus, penile cancer, squamous cell carcinoma

## Abstract

**Objectives:**

To study the characteristics of penile cancer in French Guiana (FG)— a French overseas department in South America. Indeed, penile cancer is a rare malignancy with significant geographic and socioeconomic disparities. While its epidemiology is well‐documented in mainland France, data from FG remain limited.

**Patients and Methods:**

We conducted a retrospective analysis of 22 cases of primary penile cancer diagnosed between 2004 and 2024 at the Centre Hospitalier de Kourou. Demographic, clinical, histopathological, and risk factor data were collected and reviewed.

**Results:**

The average incidence was 1.07 cases per year, with a mean age at diagnosis of 54.9 years. Notably, 19% of patients were under 40 years. The Bushinengue population (descendants of escaped African slaves) accounted for 54.5% of cases. The most common risk factor was lack of circumcision (100%), followed by HPV‐16 infection (40.9%). Most tumours were exophytic (68.2%), distal (72.7%), with a median size of 3.5 cm. Squamous cell carcinoma was the predominant histological type (90.9%), with 56.3% being well differentiated. Lymph node involvement was present in 68.2% of patients.

**Conclusion:**

The incidence of penile cancer in FG appears higher than in neighbouring regions, potentially due to regional underreporting and cross‐border healthcare access. The disproportionate impact on the Bushinengue population, younger age at diagnosis and advanced disease at presentation likely reflect cultural practices, low circumcision rates and barriers to early care. This first study on penile cancer in FG highlights the role of non‐circumcision and HPV‐16 infection as major risk factors. Public health efforts should prioritize HPV vaccination and early diagnostic access in vulnerable populations.

## 1 INTRODUCTION

Penile cancer (PCa) is a rare disease. The worldwide incidence of penile cancer is 0.95/100000 men and is thought to be rising.^1^, ^2^ Regional disparities exist in the incidence rates, with the highest rates noted in South America, South‐East Asia and parts of Africa. Penile cancer may account for 1–2% of malignant disease in men. The age‐adjusted annual incidence ranges from 0.7 to 3.0 per 100,000 population in India and 8.3 per 100,000 population in Brazil, and is even higher in some parts of Africa such as Uganda.^3^ Conversely, in industrialized countries, the overall incidence of penile cancer is around 0.94 per 100,000 men in Europe and 0.5 per 100,000 men in the USA.^3^ Although it can affect people of any age, penile cancer is often diagnosed in the sixth and seventh decades of life.^4^, ^5^ Several risk factors have been described: lack of circumcision, phimosis, poor glans hygiene, smoking, chronic inflammation, multiple sexual partners and infection with the oncogenic Human Papilloma Virus (HPV), notably HPV 16 and 18 genotypes.^6^, ^7^, ^8^ Clinically, it is a lesion of the penis, usually painless, with a budding or ulcerated appearance, mostly affecting the glans or prepuce.^3^, ^6^, ^9^

While the epidemiology of penile cancer in France is well known, the situation in French Guiana (FG), a singular French overseas department in South America, remains under‐evaluated. FG is marked by significant geographic isolation and health inequalities. Approximately 95% of its territory is covered by the Amazonian rainforest, and many communities are only accessible by river or air. These structural challenges, combined with socioeconomic difficulties, linguistic diversity, as well as challenges arising from cultural differences in healthcare, can lead to delays or renouncement in accessing specialized medical care. This situation can represent a major barrier to early cancer diagnosis and likely contributes to advanced stages at presentation.^10^ Furthermore, HPV prevalence is much higher than in mainland France.^11^ Given this context, we hypothesize that the epidemiology of penile cancer in FG diverges from mainland France. We thus hereby describe the epidemiological, histopathological and clinical aspects of penile cancer in FG.

## 2 PATIENTS AND METHODS

This is a retrospective study of 22 cases of primary penile cancer identified over a period of 20 years between 2004 and 2024 in the urology department of the Centre Hospitalier de Kourou in FG, which is the only urology department in FG. The parameters studied were: age, phenotype, risk factors, duration of evolution, clinical aspects of the lesion and histological types.

Regarding diagnosis of lymph node involvement, the diagnosis was based on both clinical and radiological findings: clinical: 8/22 (36,4%), radiological: 15/22 (68,2%).

18/22 patients had an abdomen and pelvis CT scan with evaluation of the inguinal region. 1/22 patient had a TEP 18‐FDG scan.

Imaging modality employed for metastasis evaluation was according to the contemporary French Association of Urology guidelines and included an abdomen‐pelvis CT scan with chest evaluation in case of fixed inguinal or pelvic lymph nodes (cN3).

## 3 RESULTS

In total, 22 patients were included in our study. The annual incidence was 1.07 cases/year.

The mean age at diagnosis was 54.95 years (23–86 years), and 19% of patients were younger than 40 at diagnosis. The Bushinengue were the most affected population at 54.55% (Guyanese Bushinengue 36.36%, Surinamese Bushinengue 18.18%), followed by Brazilians (13.64%) and Guyanese Creoles (13.64%) (Table 1).

**TABLE 1:** Phenotype/ethnicity.

Phenotype/ethnicity	% (N)
Guyanese Bushinengue	36,36% (8/22)
Surinamese Bushinengue	18,18% (4/22)
Brazilian	13,64% (3/22)
Creoles	13,64% (3/22)
Caucasian	9,09% (2/22)
Haitian	9,09% (2/22)

Among the risk factors identified, lack of circumcision was the most common (100%) followed by HPV‐16 infection (40.90% ‐ 9/22), phimosis (13.64% ‐ 3/22) and tobacco use (13.64% ‐ 3/22).

**FIGURE 1:**
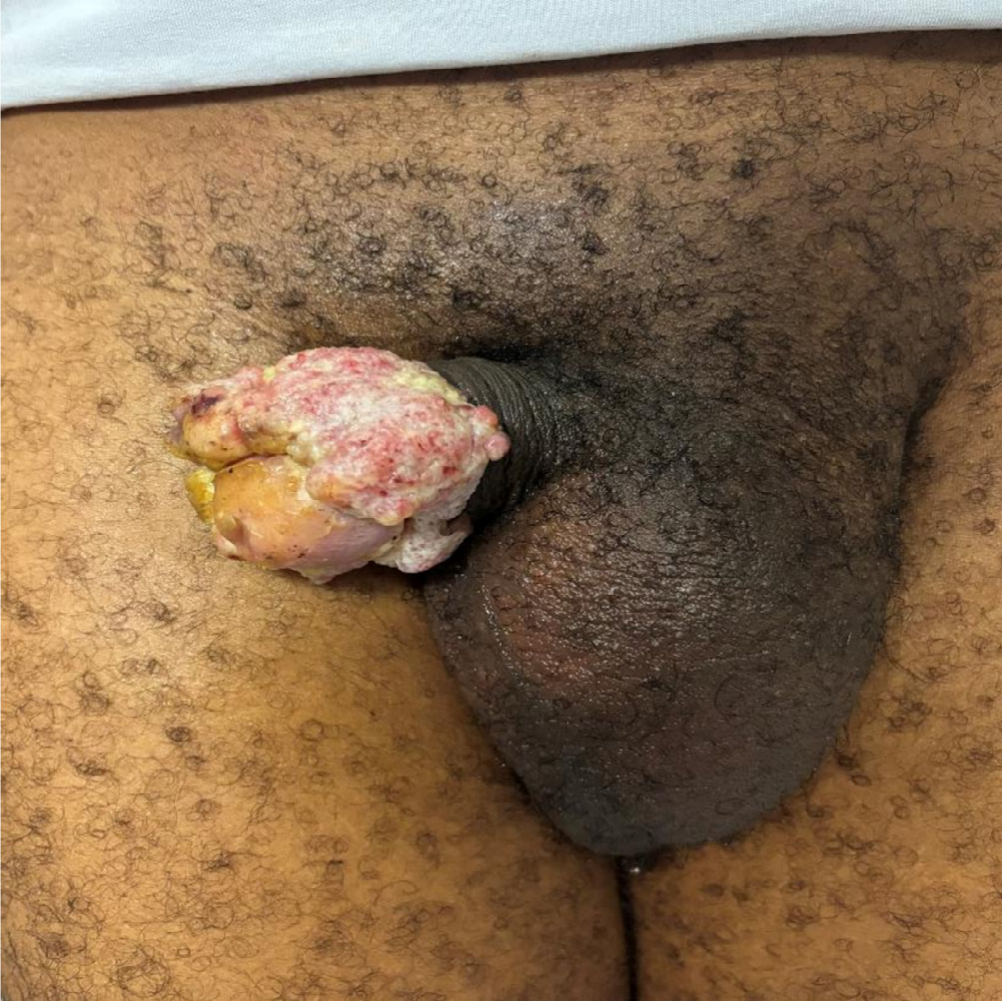
Exophytic lesion of the glans and balanopreputial sulcus.

**FIGURE 2:**
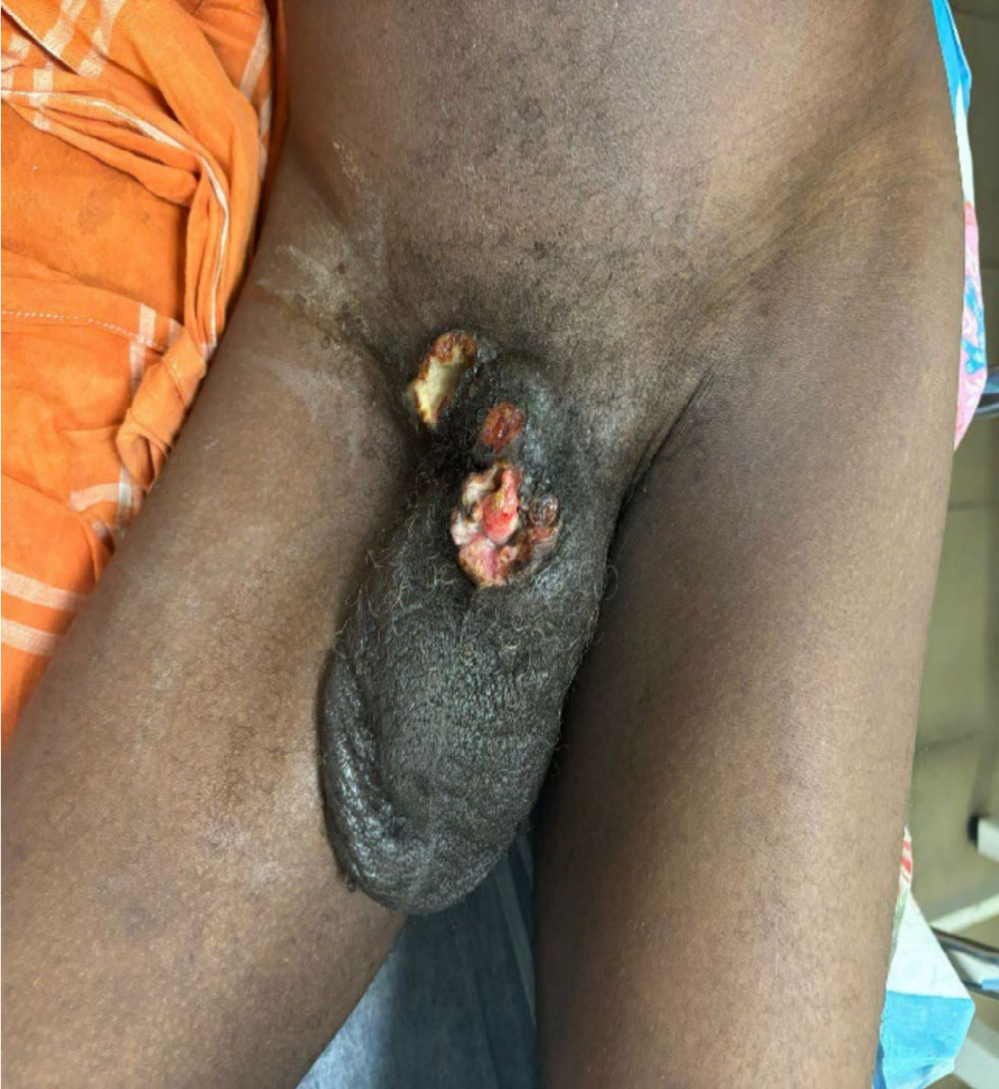
Complete self‐amputation of the penis by an ulceronecrotic penile lesion associated with a right inguinal lymph node ulceration.

The average interval between lesion onset and the first medical consultation was 11 months (range: 4–24 months). All patients presented with a visible penile lesion, either exophytic (68.18%) or ulcerative (31.82%) in nature (Figure 1). The median lesion size was 3.5 cm (range: 1–10 cm). Notably, one patient exhibited complete self‐amputation of the penis due to the lesion (Figure 2). The initial surgical treatment consisted of 1. Regarding surgical penile treatment: glansectomy: 3/22 (13.6%), partial penectomy: 7/22 (31.8%), total penectomy: 5/22 (22.7%), radiation therapy/brachytherapy: 2/22 (9%), posthectomy: 2/22 (9%), biopsy and chemotherapy: 2/22 (9%), biopsy but lost to follow‐up: 1/22 (4.5%). 2. Regarding inguinal lymph node dissection (ILND): bilateral ILND: 6/22 (27.3%), unilateral ILND: 1/22 (4.5%) and chemotherapy was used for 6 out of the 15 patients who had N + disease.

The majority of lesions (72.73%) were located distally: on the prepuce in 13.64% (3/22), the glans in 13.64% (3/22) and the glans with involvement of the balano‐preputial sulcus in 45.45% (10/22). The penile shaft was involved in only 27.27% of the cases (6/22).

The majority of penile cancers in our study were squamous cell carcinomas (90.90%), including 9 cases (40.90%) induced by HPV‐16 and 2 cases (9.09%) associated with Bowen's disease. One patient was diagnosed with a pseudomyogenic hemangioendothelioma, initially misidentified as a squamous cell carcinoma of the penis on biopsy. Among the squamous cell carcinomas, 56.25% were well differentiated (G1) (Table 2). Clinically, T1 was the most common stage, observed in 45.46% of cases, and lymph node involvement was present in 68.18% of patients (Table 3).

**TABLE 2:** Histological characteristics.

Histological characteristics	% (N)
**Histological type**	
Squamous cell carcinoma	90,90 (20/22)
High‐grade PIN	4,55 (1/22)
Pseudo‐myogenic hemangioendothelioma	4,55 (1/22)
**Histological grade**	
G1(Well differentiated)	56,25 (9/16)
G2 (Moderately differentiated)	37,50 (6/16)
G3 (Poorly differentiated)	0 (0/16)
G4 (Undifferentiated)	6,25 (1/16)

**TABLE 3:** cTNM according to classification of penile cancer (OMS 2016).

	% (N)
**T stage**	
T1	45,45 (10/22)
T2	36,36 (8/22)
T3	13,64 (3/22)
T4	4,55 (1/22)
**Node involvement cN**	
N0	31,82 (7/22)
N1	0 (0/22)
N2	59,09 (13/22)
N3	9,09 (2/22)
**Metastasis M**	
M0	95,45 (21/22)
M1	4,55 (1/22)

Three patients had disease recurrence at one year follow‐up. We have no further data regarding long‐standing cure as much of the patients are lost to follow‐up, living in remote and hard‐to‐reach areas of FG.

## 4 DISCUSSION

Penile cancer is one of the least commonly encountered cancers in the routine practice of urologists. Over a 20‐year period, 22 cases of penile cancer were diagnosed and managed at the Urology Department of the Kourou Hospital Center in FG, with an incidence of approximately 1.07 cases per year. According to the International Agency for Research on Cancer (IARC) in 2022, the age‐standardized incidence of penile cancer in FG is 1.5/100000 men. This incidence is higher than that observed in South America (1.4/100000 men), Suriname (1.4/100000 men) and Brazil (1.3/100000 men).^1^ This difference may be explained by the fact that populations from neighbouring countries (Suriname and Brazil), seeking optimal healthcare and social services, migrate to FG and are treated in the hospitals of FG. Our results may support this, as 13.63% (3/22) and 18.18% (4/22) of our patients were Brazilian and Surinamese, respectively. However, the IARC report only included six Brazilian cities (Aracaju, Belo Horizonte, Cuiabá, Fortaleza, Goiânia and São Paulo) in calculating the national incidence. Most of these cities (four out of six) are not in the poorest regions of Brazil, where penile cancer is more common, suggesting that the data may not accurately reflect the incidence of the disease in Brazil. Several studies, such as those by Fu et al. and Favorito et al., report much higher age‐standardized incidence rates of 8.3/100000 and 2.8–6.8/100000, respectively, in Brazil.^3^, ^12^

The average age at diagnosis of penile cancer in our study was 54.95 years, with 19% of patients being under 40 years of age. This is relatively younger than the mean age of 71 years reported in metropolitan France,^13^ but comparable to the 58.6 years reported by Coelho et al. in Maranhão, Brazil,^14^ where 20.8% of patients were under 40. Both FG and Brazil have predominantly young populations and face higher rates of unemployment and poverty compared to European countries. In 2023, Brazil's unemployment rate was 7.4%, with a poverty incidence of 5.2% based on the national poverty line.^15^ FG has experienced persistently high unemployment rates, ranging between 17% and 24% over recent decades, and significantly higher poverty levels compared to metropolitan France.^16^ These socioeconomic factors may contribute to earlier exposure to penile cancer risk factors and a younger average age at onset.

Risk factors for penile cancer include: lack of circumcision, phimosis, poor hygiene of the glans, chronic inflammation, smoking, multiple sexual partners, Human Papillomavirus (HPV) infection and low socioeconomic status.^6^, ^7^, ^8^ The pathophysiology of penile cancer remains poorly understood. Lack of circumcision was the most commonly observed risk factor in our study (100%), followed by HPV‐16 infection (40.90%), phimosis (13.63%) and smoking (13.63%). Several authors report a strong association between the presence of the foreskin and the development of penile cancer.^17^ The risk of developing penile cancer was studied by Maden et al.^17^ in three groups of individuals. Among those who had never been circumcised, the risk was 3.2 times higher than in those circumcised at birth and 3 times higher than in those circumcised during the neonatal period. Similarly, penile cancer incidence is low in countries like Israel (0.3/100000), where neonatal circumcision is routinely performed for cultural reasons.^2^ Neonatal circumcision reduces the risk of penile cancer; however, the protective effect of circumcision performed in adulthood remains a subject of debate. One notable risk factor associated with the presence of the foreskin is phimosis, a condition characterized by the inability to retract the foreskin. This can lead to the accumulation of smegma, a mixture of skin cells, bodily fluids and microorganisms, creating an environment conducive to inflammation and potential carcinogenesis.^18^ Poor hygiene practices exacerbate this environment, leading to chronic irritation and inflammation that may contribute to the initiation and progression of penile cancer.^19^ Nine cases (40.90%) of HPV‐16 were diagnosed through biopsies. HPV is known to be associated with penile cancer in more than one‐third of cases, with genotypes HPV 16 and 18 being the most frequent.^2^, ^7^, ^8^ In Maranhão, Brazil, HPV‐16 is found in 80.5% of penile cancers,^7^ which differs from our findings. This difference could be explained by the small sample size in our study. HPV status has prognostic significance, as HPV‐positive penile cancers tend to have a more favourable prognosis than their HPV‐negative counterparts.^20^, ^21^, ^22^ In France, the High Authority for Health (HAS) and the Cancer Committee of the French Urological Association recommend HPV vaccination for all boys aged 11 to 14 years, with a catch‐up vaccination possible for those aged 15 to 19 years, to prevent HPV‐related cancers (penis, anus, oropharynx), and to also improve protection for unvaccinated girls (cervix, anus, oropharynx).^4^ Smoking, a well‐established risk factor for various cancers, is implicated in the aetiology of penile cancer as both a direct and independent risk factor related to dose.^23^ Tsen et al., in their case–control study, showed a 2.4‐fold increase in risk among those who had smoked and an even higher incidence in current smokers.^24^

The Bushinengue population was the most affected, accounting for 54.55%. The population of FG is predominantly (60%) of African and Afro‐Caribbean descent (Creole Guianese, Caribbean Creoles and Bushinengue), but also includes metropolitan French (8.07%) and other populations such as Chinese, Lebanese, Brazilian, Haitian, etc.^25^

The Bushinengue (Djukas, Saramacas, Bonis, etc.) are descendants of escaped slaves from Suriname, residing along rivers and within the Amazonian forests, which encompass approximately 95% of FG's territory. They maintain strong traditions of medicinal plant use, often collecting and preparing remedies within their communities.^26^ Cultural practices, including polygamy and a general absence of circumcision, may contribute to their heightened vulnerability to penile cancer observed in our study. Additionally, limited access to healthcare—due to geographic isolation, underdeveloped transportation infrastructure and linguistic or cultural barriers—can delay medical consultation. Such delays are common among remote populations in FG and are associated with more advanced disease presentations.^10^ Consequently, patients are often seen with significantly progressed lesions, exemplified by a case of complete penile self‐amputation due to tumour progression (Figure 2) and reflected by the average lesion size of 3.4 cm and a predominance of locally advanced stages (68.18%).

The mode of presentation of penile cancer in all patients was the presence of a macroscopic lesion, either exophytic (68.18%) or ulcerative (31.82%) on the penis. These lesions were distal (prepuce and glans) in 72.73% of cases. This mode of presentation is described by several authors.^2^, ^6^, ^9^ The majority of penile cancers in our study were squamous cell carcinomas (90.90%). According to the literature, squamous cell carcinoma of the penis represents 95% of primary malignant tumours of the penis, followed by rare forms such as melanomas and sarcomas.^2^, ^6^ Squamous cell carcinomas of the penis can be subclassified into several histological variants depending on their association with HPV (usual, verrucous, basaloid, papillary, sarcomatoid and mixed types). Mixed forms, including verrucous and papillary components, have a favourable prognosis, while basaloid, adenosquamous and sarcomatoid forms tend to have a poor prognosis.^27^ These squamous cell carcinomas were well‐differentiated (G1) in 56.25% and moderately differentiated (G2) in 37.50%, with penile cancer generally being well‐differentiated (G1) or moderately differentiated.^23^ One case of pseudomyogenic hemangioendothelioma was reported in our study, initially mistaken for a squamous cell carcinoma of the penis on biopsy. Pseudomyogenic hemangioendothelioma is a very rare, recently described vascular tumour with intermediate malignancy, predominantly affecting soft tissues in the limbs and occurring at a young age, typically in males.^28^

## 5 CONCLUSION

This study provides the first comprehensive epidemiological, clinical and histopathological characterization of penile cancer in FG, a region previously underrepresented in global cancer registries. With a relatively high incidence compared to neighbouring countries, a younger average age at diagnosis and a predominance of cases among marginalized populations such as the Bushinengue. Our findings highlight the critical impact of cultural practices, socioeconomic disparities and limited healthcare access on disease burden and presentation. By identifying the prominent role of non‐circumcision and HPV‐16 infection, this study also reinforces the need for targeted public health interventions, including culturally sensitive education, early detection programs and the implementation of HPV vaccination. These insights fill an important knowledge gap and may inform both local and regional strategies to reduce the morbidity and mortality associated with penile cancer in similar underserved populations.

## AUTHOR CONTRIBUTIONS

*Lead writing author*: Khalil Chalhoub. *Data collection*: Bawa Nonoa. *Pathology expertise*: Vincent Molinie. *Manuscript review*: Vincent Ravery.

## CONFLICT OF INTEREST STATEMENT

The authors declare no conflicts of interest.
